# MEK and TGF-beta Inhibition Promotes Reprogramming without the Use of Transcription Factor

**DOI:** 10.1371/journal.pone.0127739

**Published:** 2015-06-03

**Authors:** Jan Vrbsky, Tamas Tereh, Sergiy Kyrylenko, Petr Dvorak, Lumir Krejci

**Affiliations:** 1 Department of Biology, Faculty of Medicine, Masaryk University, Kamenice 5, 625 00, Brno, Czech Republic; 2 International Clinical Research Center, St. Anne’s University Hospital Brno, Pekarska 53, 656 91, Brno, Czech Republic; 3 National Centre for Biomolecular Research, Faculty of Science, Masaryk University, Kotlarska 2, 602 00, Brno, Czech Republic; University of Newcastle upon Tyne, UNITED KINGDOM

## Abstract

The possibility of replacing the originally discovered and widely used DNA reprogramming transcription factors is stimulating enormous effort to identify more effective compounds that would not alter the genetic information. Here, we describe the generation of induced pluripotent stem cells (iPSc) from head-derived primary culture of mouse embryonic cells using small chemical inhibitors of the MEK and TGF-beta pathways without delivery of exogenous transcription factors. These iPSc express standard pluripotency markers and retain their potential to differentiate into cells of all germ layers. Our data indicate that head-derived embryonic neural cells might have the reprogramming potential while neither the same primary cells cultivated over five passages *in vitro* nor a cell population derived from adult brain possesses this capacity. Our results reveal the potential for small molecules to functionally replace routinely used transcription factors and lift the veil on molecular regulation controlling pluripotency. The conditions described here could provide a platform upon which other genome non integrative and safer reprogramming processes could be developed. This work also shows novel potential for developing embryonic neural cells.

## Introduction

A majority of techniques routinely used for reprogramming induced pluripotent stem cells (iPSc) utilize direct delivery of selected transcription factors (TFs). The most critical factors are Oct4, Sox2, Nanog, c-Myc, Klf4 and Lin28 [1–5]. Since the initial reprogramming experiment by Yamanaka [5], a number of other genes and factors contributing to the reprogramming process have been identified [6]. Current reprogramming methods are mostly based on delivery of reprogramming TFs in the form of exogenous DNA. Other methods use mRNA forms of TFs [7,8], micro-RNAs [9,10], or purified recombinant TFs with the help of other enhancers like valproic acid [11,12]. In certain cell types, some reprogramming TFs can be dispensed with due to those cells’ relatively high endogenous expression levels [13,14] or these can be substituted by other components [15,16].

Chemical inhibition of mitogen-activated protein kinase (ERK1/2) [17,18] and glycogen synthase kinase 3 (GSK3) pathways has been shown to significantly increase efficiency of reprogramming [19]. Additionally, these inhibitors were sufficient to substitute for LIF and BMP signaling, important for maintaining pluripotency and preventing differentiation in mouse embryonic stem cells (ESc) and iPSc [20,21]. Inhibition of both MEK and ERK1/2 have been shown to increase efficiency of reprogramming by Klf4 or by Oct4 and Klf4 [22,23], indicating that targeting both TGF-beta and MEK signaling pathways might help with reprogramming. Highly efficient reprogramming has also been achieved by dual inhibition of MEK (PD0325901 inhibitor) and GSK3 (CHIR99021 inhibitor) in partially reprogrammed iPSc derived from neural stem cell (NSc) upon transduction with Oct4 and Klf4 [22,23]. Moreover, induction of endogenous Nanog by inhibition of TGF-beta (RepSox inhibitor) has been reported to be sufficient to replace Sox2 from the reprogramming cocktail [24]. Meanwhile, the use of exogenous transcription factors has been substituted completely by using a cocktail of seven small-molecule compounds which were sufficient to reprogram pluripotent stem cells from mouse somatic cells [25].

## Results and Discussion

### Reprogramming of primary cell cultures with TGF-beta and MEK inhibitors

Numerous molecular components of TGF-beta and MEK signaling pathways seem to be involved in regulation of pluripotency or differentiation. Inhibition of TGF-beta could lead to inactivation of SMAD, which is needed for BMP signaling toward differentiation [26]. Additionally, ERK1/2 has been shown to inhibit Nanog expression [27], and therefore its inhibition might have a positive effect on transition toward pluripotency.

**Fig 1:**
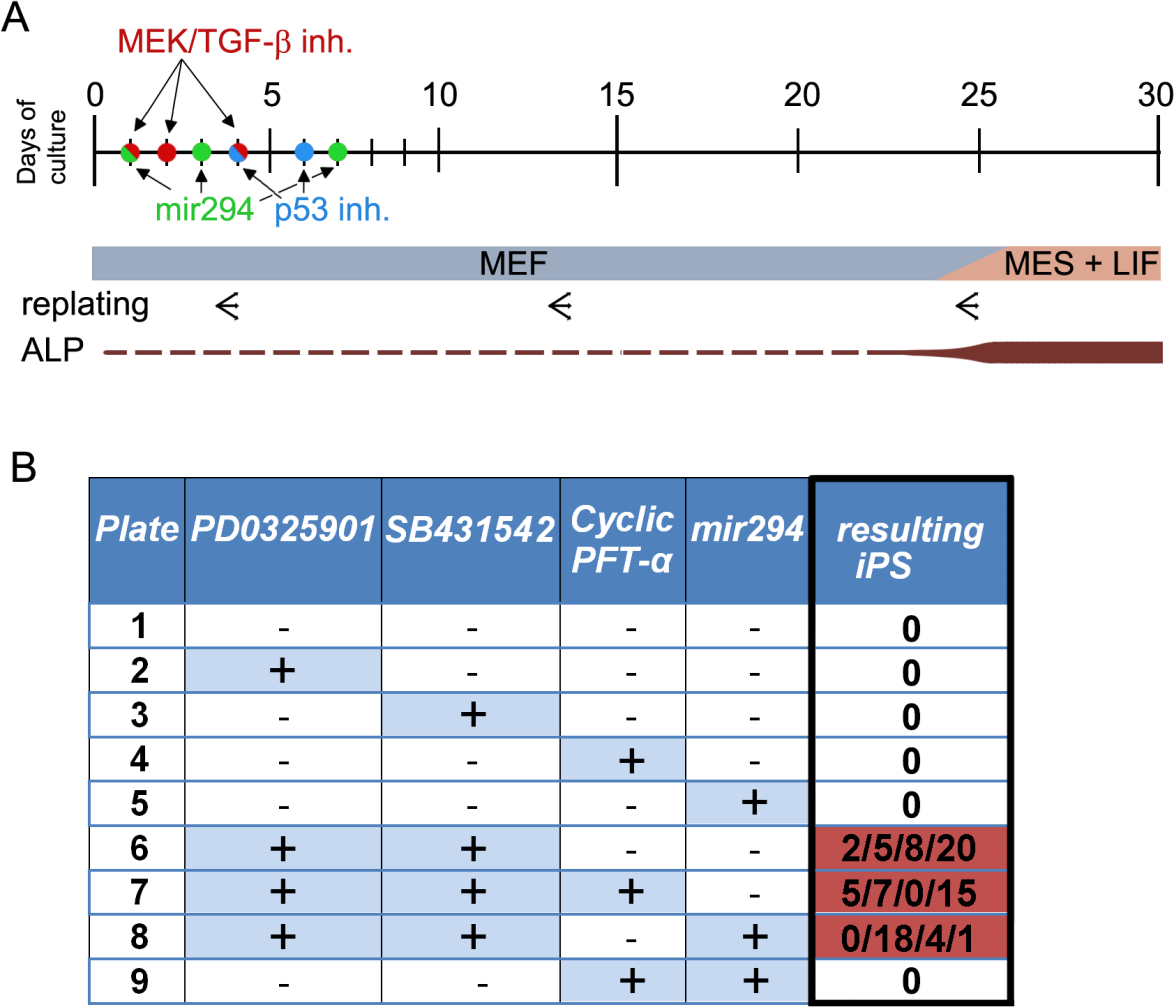
Timescale of the reprogramming experiment and combinations of inhibitors. **[A]** Cell culture was seeded at concentration of 25,000–33,000 cells/cm^2^ at day 0 (1.7 x 10^6 cells per 10cm dish). On days 1, 2 and 4 the inhibitors PD0325901 and SB431542 were applied at concentrations 0.5 μM and 2 μM. Cultures were three times replated (1:3 ratio) as indicated by a triple arrow. At day 25, the first colonies positive for alkaline phosphatase were detected (brown bar) and MEF culture medium was replaced by LIF–containing ESM medium (blue bar). In parallel experiments, fibroblast cultures were treated with mir294 mimics (days 1, 3 and 7) and p53 inhibitor (days 4 and 6). **[B]** Combinations of inhibitors for chiPSc reprogramming as applied on individual plates (experimental setup 1–9) with the number of resulting morphologically distinguishable and phosphatase positive iPSc-like colonies from four independent experiments in each setup.

**Fig 2:**
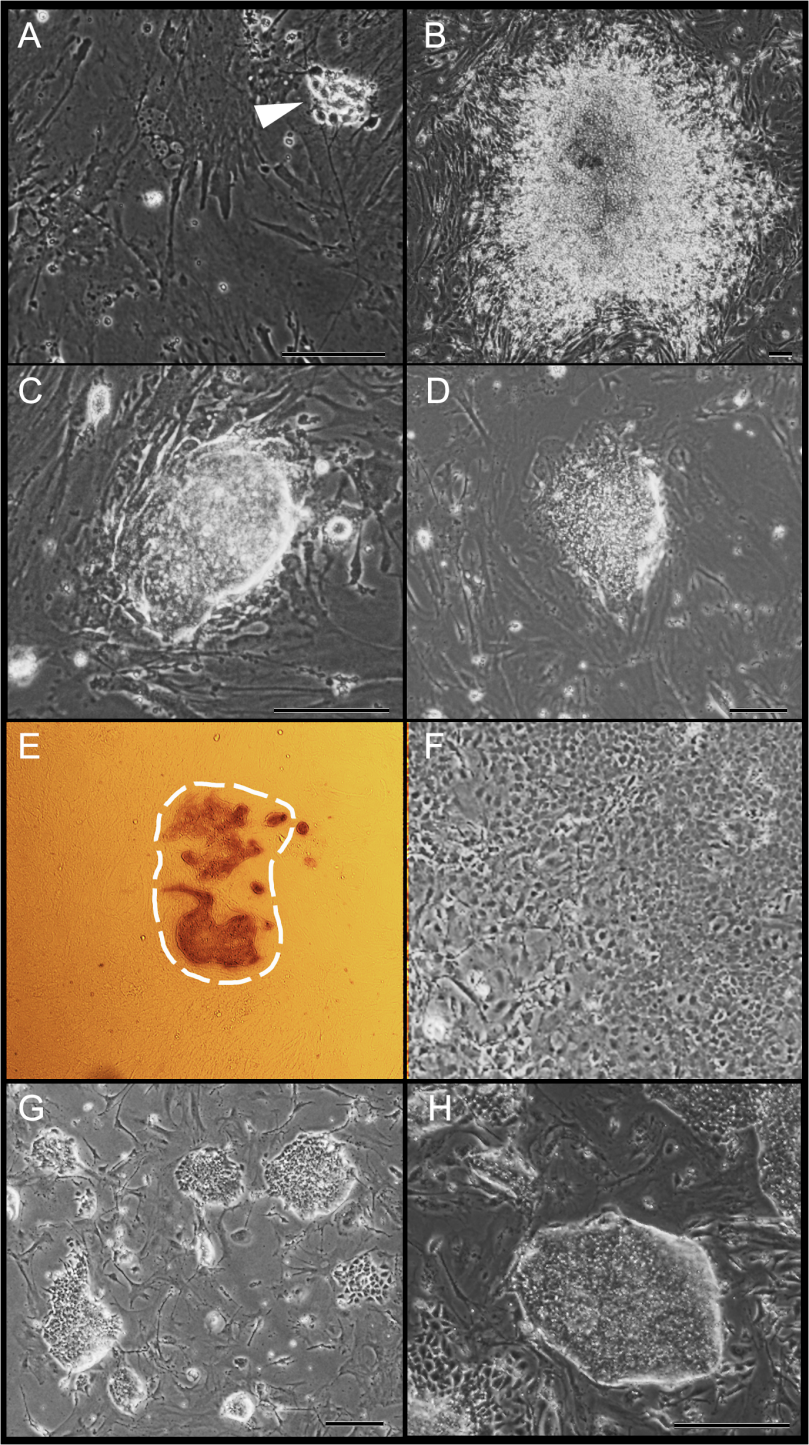
Treatment with small-chemical inhibitor results in reprogramming to chiPSc. Light microscopy analysis of freshly forming chiPSc colonies from four independent experiments. Day 0 of culture **[A]**, with bipolar cells indicated by an arrow. First pre-iPSc colonies **[B–D]** at day 25 of culture treated with inhibitors after second replating on mouse feeder layer. The immunostaining for cells positive for alkaline phosphatase at day 21 of culture with inhibitors; shape of the colony is outlined **[E]**. Day 28 of culture without treatment by inhibitors **[F]**. chiPS colonies with their characteristic morphology **[G, H]**. Scale bars in all figures are 10 μm.

In order to test whether chemical inhibitors of TGF-beta and MEK signaling pathways are sufficient for reprogramming towards pluripotency, primary cell culture from CF-1 mouse embryo at 12.5 days post coitum (DPC) was treated repeatedly within 5 d with chemical inhibitors of MEK (PD0325901; 0.5 μM) and TGF-beta (SB431542; 2 μM) (See Fig 1A and Materials and Methods section for details). To further increase the efficiency of reprogramming, we also added in parallel previously published enhancers of reprogramming, namely microRNA mimics [9,28,29] and inhibitor of p53 (cyclic PFT-alpha) [30]. Over the next 4 weeks, the cell cultures were examined daily for iPSc colony formation. At the end of week 4, from 0 to 20 morphologically distinguishable iPSc-like colonies positive for alkaline phosphatase were detected (Figs 1B and 2). As shown in Fig 1B (highlighted in red), iPS-like colonies formed only in samples treated with both MEK and TGF-beta inhibitors. No alkaline phosphatase-positive colonies were detected when a single treatment of MEK, TGF-beta and p53 inhibitors or mir294 mimics [29] was applied. Nor was significant increase in the number of colonies observed with addition of mir294 mimics and PFT-alpha into the plates treated with MEK and TGF-beta inhibitors. This indicates that just the combination of inhibitors is sufficient to achieve reprogramming. Finally, individual morphologically distinguishable colonies were manually passaged and expanded on MEF-feeder-layer dishes with ESM medium.

### Chemically reprogrammed iPS lines (chiPS) display markers of pluripotency

**Fig 3:**
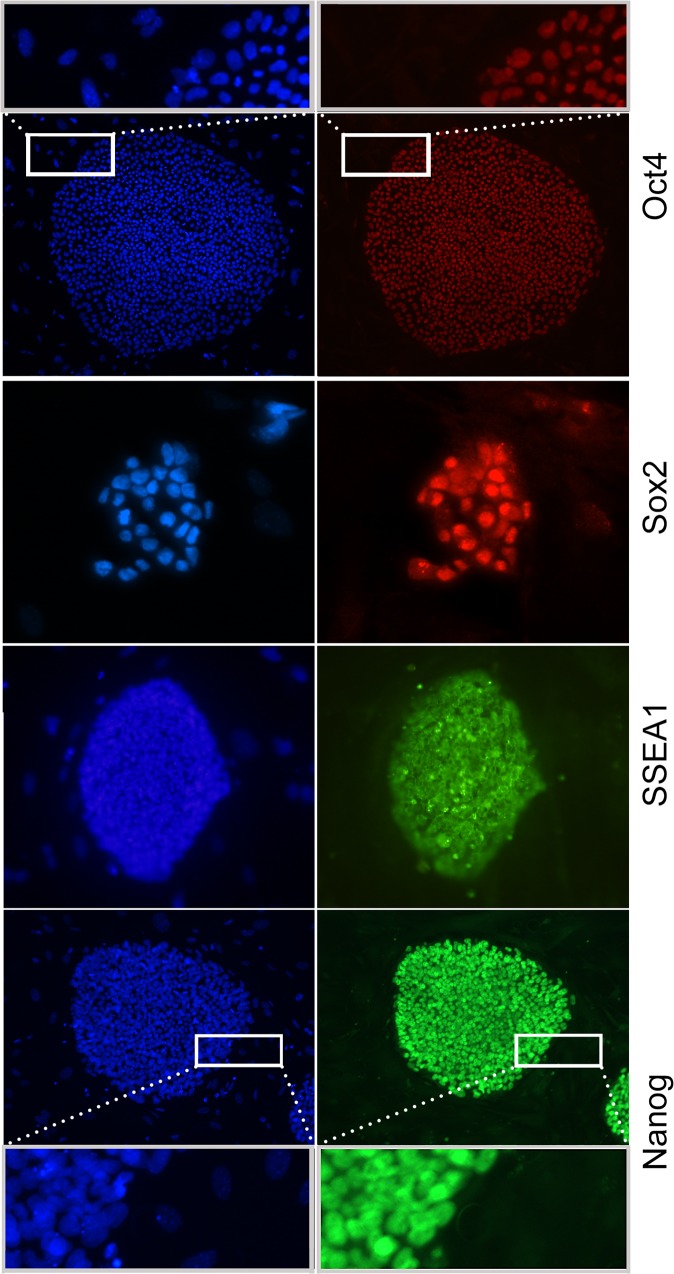
Expression of pluripotency markers in chiPSc colonies. Left column: staining of nuclei using DAPI (blue). Right column: the same colony stained with antibodies against several pluripotency markers including, Oct4, Sox2, Ssea1 and Nanog. Oct4- and Nanog-positive colonies are enlarged to show specificity of the staining in comparison to surrounding fibroblasts. 100x magnification was used for Oct4, Ssea1 and Nanog, 200x magnification for Sox2.

Pluripotency potential of propagated colonies was tested using immunocytochemistry to detect typical pluripotency markers, including Nanog, Sox2, SSEA1 and Oct4. As shown in Fig 3, corresponding pluripotency markers were detected indicating successful reprogramming. The estimated efficiency of reprogramming was about 4 iPS-like colonies per 1.7 x 10^6^ starting cells. To determine how many of these colonies will show pluripotency markers, we analyzed 35 iPS-like colonies derived from 12.5 DPC embryos. Approximately 40% of clones were AP positive and showed linage stability over 7 passages (data not shown). Cell lineage stability of two individual chiPS lines was further analyzed by their propagation for more than 5 months (> 30 passages) in ESM medium with no obvious reduction of cell viability and expression of pluripotency markers. Karyotype analysis of early passage chiPS (passage number 4) revealed their normal karyotype (S1 File), even though we also observed abnormal number of chromosomes in three other lines (at passage number 27, 28 and 55; modal chromosomal number 38, 42 and 50, respectively). Similar observation describing chromosomal instabilities of mouse pluripotent cells (both iPS and ES) was described previously [31].

**Fig 4:**
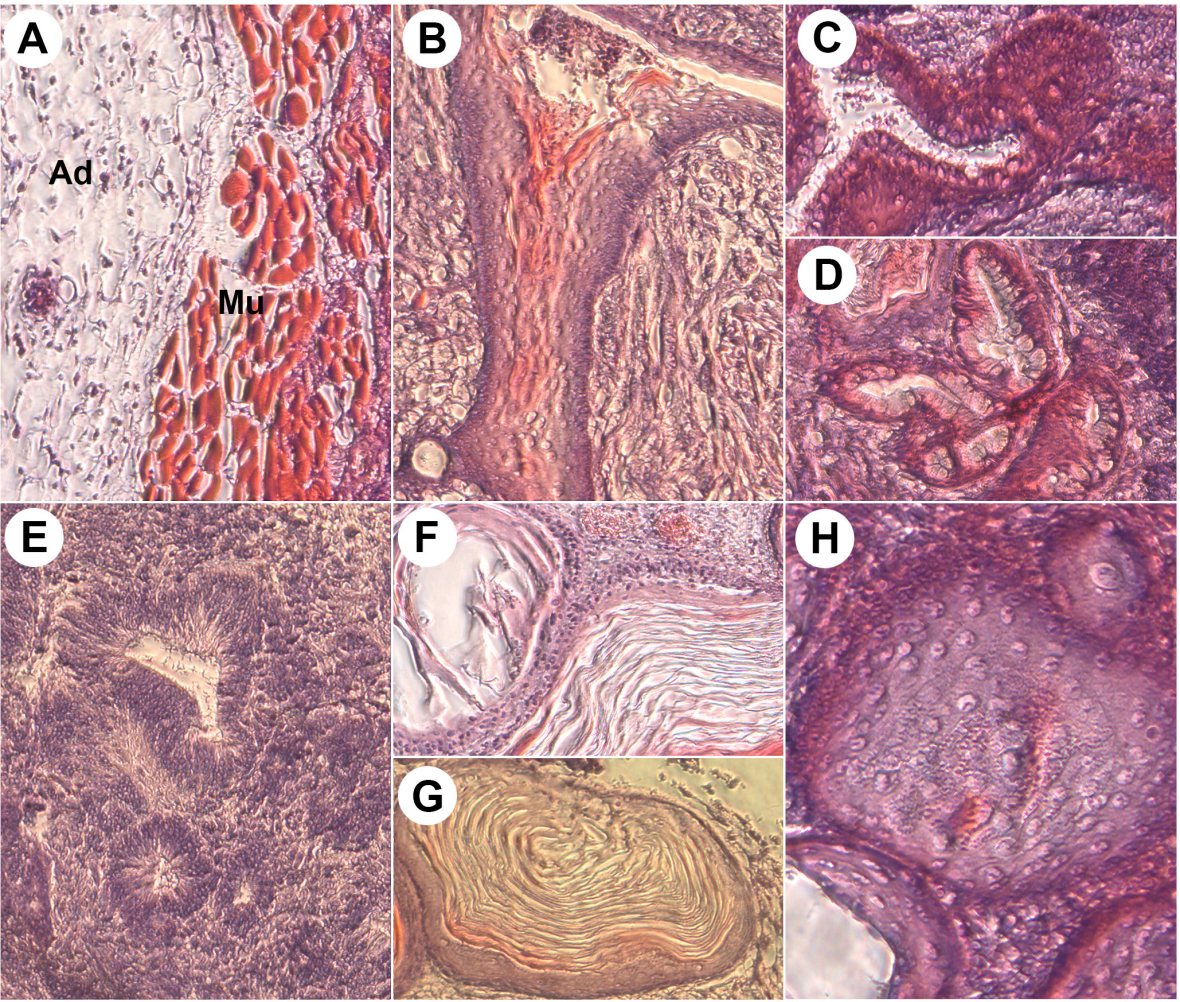
Histological characterization of chiPSc-derived teratomas. Two independent chiPSc lines (two replicas of each line) were tested in nude mice. Histological sections of hematoxylin-and-eosin-stained sections of teratomas from both lines consist of tissues from all three germ layers. A) Adipose-like tissue [Ad], muscle [Mu]; B) bone; C,D) gut-like epithelial structures with mature goblet cells; E) neuroblastic tissue with neuronal rosettes; F,G) epidermis; and H) cartilage. Magnified 100x.

To test whether these chiPS can differentiate into cells of all germ layers, we performed teratoma formation assay on two different chiPS lines. Both lines gave rise to cells of all the embryonic layers, thus indicating their pluripotency potential (Fig 4). We further tested the specific neural differentiation potential of chiPS using neural induction serum-free medium (N2B27) according to published protocol [17,32]. One week after treatment with N2B27, the chiPS readily differentiated into neurons as confirmed by detection of neural markers Tuj1 and Nestin (S2 File). Taken together, chiPS derived by inhibition of TGF-beta and MEK pathways express several characteristic pluripotency markers and maintain the ability to differentiate into neurons as well as other cell types.

### Specificity of different primary cell cultures to the TGF-beta and MEK inhibitors

To explore more specifically which cell types of the embryos respond to TGF-beta and MEK inhibitors and give rise to chiPS, we tested five other primary cell cultures derived from liver, side-body skin, head, brain and tail-tip of the embryo. As a control, we used previously tested whole-embryo-derived cell culture (see methods). These samples were treated using the same reprogramming protocol, which led to chiPS as described previously. Neural samples derived from purified brain section did not proliferate in the given culture conditions, as they require defined and serum-free culture conditions [33], and thus these were not utilized in subsequent experiments. All the other samples formed adherent cell cultures with sufficient proliferative speed and cell viability (S3 File). Four weeks later, we detected several chiPS colonies (2–10 in four independent experiments) expressing standard pluripotency markers only in those samples derived from head parts (for simplicity’s sake, we refer to these cells as HDC: head-derived cells) or from whole embryos (Fig A in S4 File). Similarly to the previous reprogramming experiments, chiPS colonies were observed only when both the TGF-beta and MEK inhibitors were used (Fig 2G and 2H).

In the developing embryo, there is a cell-specific population of epiblast-derived stem cells (EpiSC) which resemble ESc in many characteristics including pluripotency markers and colony morphology [34,35]. Additionally, this population of EpiSC was recently reported to be reprogrammable into iPS using only exogenous Klf4 and dual inhibition of MEK and ERK1/2 in medium containing LIF [22]. Although cells prone to reprogramming by our protocol were obtained from parts of the body topologically distinct from the region of the forming gonads (the source of primordial germ cells [PGCs]), we performed side-by-side immunostaining of chiPS, PGCs (*in vivo* derivatives of post-implantation epiblast cells), ESc, and iPSc lines derived using lentiviruses with four TFs (Oct4, Sox2, Klf4 and c-Myc) (S5 File). Consistent with previously published findings [36], PGCs, chiPS, iPS and ESc expressed three of the tested pluripotency markers, Nanog, Stella and Fragilis. In these experiments, however, not all morphologically distinct chiPS colonies were positive for Stella and Fragilis (data not shown), suggesting heterogeneity of the iPS-like colonies. PGC-specific marker Ddx4 [37] was observed only in epiblast-derived cells and not in chiPS, iPS or ESc, thus indicating that our chiPS did not result from expansion of EpiSC or their derivatives [34] during primary cell culture derivation.

### Developmental stage influences reprogramming by TGF-beta and MEK inhibitors

To examine whether the reprogramming capacity of the HDC changes with the developmental stage of the embryo, we compared reprogramming sensitivity of primary HDC cultures derived from 8 to 15.5 DPC and of adult mouse. While samples from 8 to 13.5 DPC resulted in generating several chiPS colonies, no reprogramming was observed from 15.5 DPC samples and those derived from adult mouse (Fig B in S4 File). To further characterize initial cell population, we propagated primary HDC *in vitro*. After 3 weeks of cultivation, we observed complete loss of reprogramming capacity (Fig A in S6 File). This might be explained by loss of neural cells from the cell culture in MEF media which is not supportive for the growth of neural cells [38]. Taken together, our data suggest that only certain embryonic developmental stages of HDC are susceptible to reprogramming by this method and these cells quickly lose reprogramming capacity when cultivated *in vitro*. This could correspond to the enormous progress in the brain development by day 15.5 results in forming the majority of neural specific structures, which is followed by *in vivo* cell-specific differentiation [39] and might explain why application of the same inhibitors on the brain-derived culture at embryonic day 15.5 did not result in generation of chiPSc colonies. Similarly, it might also explain loss of reprogramming capacity of HDC from earlier stages of the embryo after their prolonged *in vitro* cultivation. In addition, it could be due to loss of other supportive cell types promoting survival and growth of neural precursor cells under given cultivation conditions and which were present in the primary head-derived samples [40,41].

### Neural cell are sensitive to TGF-beta and MEK inhibition

Image analysis of neural markers (Nestin, Tuj1 and Gata4) in HDC samples (Figs B and C in S6 File) indicates that neural cells represent 10–30% of primary cells and suggests them as a possible target of reprogramming by our protocol. To further elucidate this role we monitored level of Sox2 neural marker in the HDC. Real-time PCR of Sox2 expression level during several time points of cultivation reveal its significant decrease during the period of two weeks (Fig D in S6 File). This coincides with their inefficiency for reprogramming, supporting the notion that neural cells in HDC might be responsible for reprogramming.

**Fig 5:**
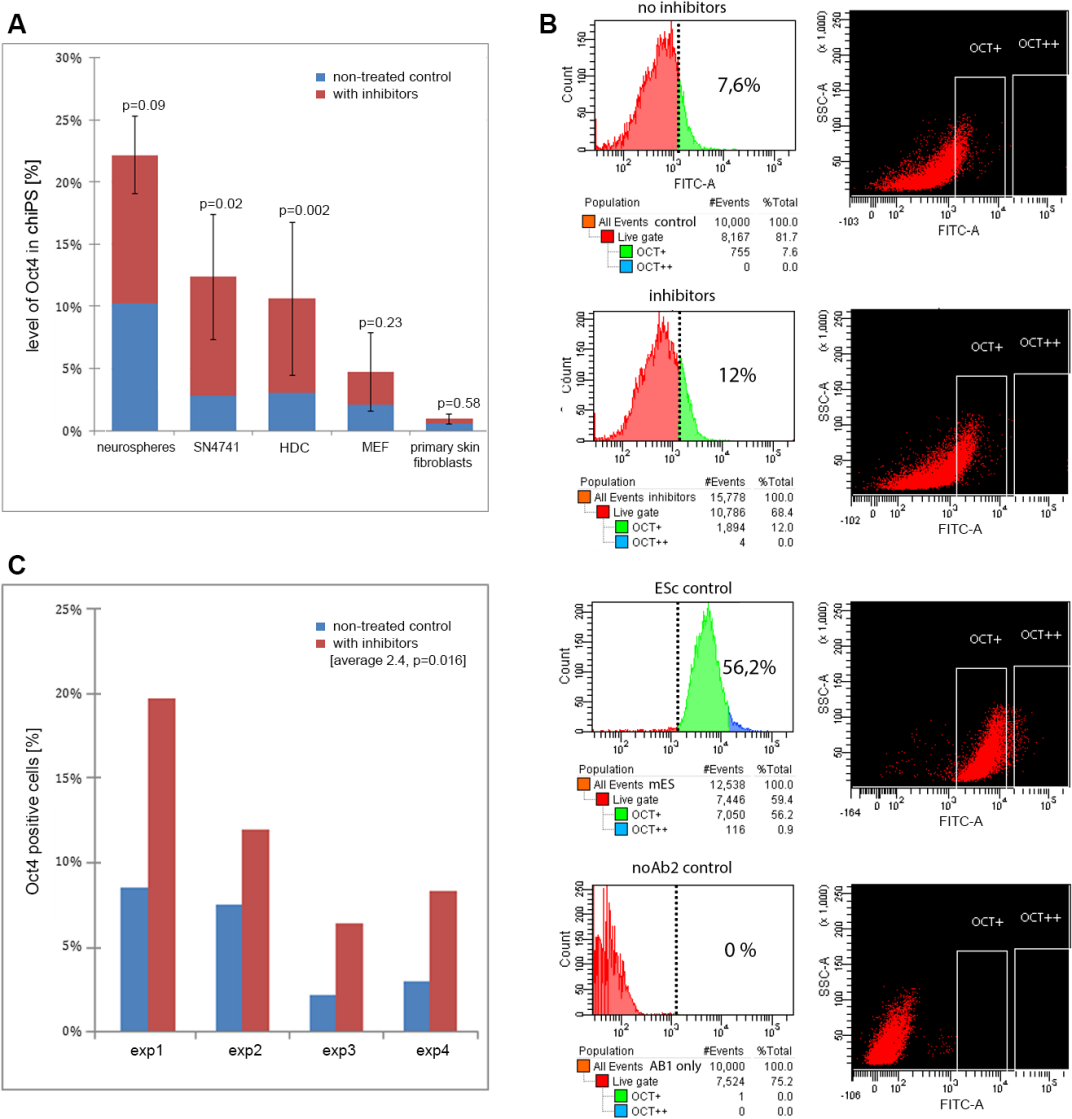
Increase of Oct4 expression after treatment with TGF-beta and MEK inhibitors. A) Oct4 expression profile by REAL-TIME PCR. Average basal Oct4 expression in treated (red) and nontreated cells (blue). Data normalized to expression of Oct4 in chiPS-derived lines (100%). While in neurospheres, the SN4741 line and HDC the expression increase is statistically significant, in the fibroblast line of later passage (MEF) and in the sample of primary skin fibroblasts the increase of Oct4 is insignificant. *P*-values for each cell type are indicated. B) FACS analysis of Oct4 expression in four different samples (from top to bottom): controls without inhibition (7.6% OCT4-positive cells), inhibitors-treated sample (12%), embryonic stem cell control on feeder layer cells (56.2%) and negative controls without secondary antibody (0%). Two gates (OCT+ and OCT++) in all experiments are indicated. We detected no distinct Oct4-positive cell population instead of global shift in the expression towards a higher intensity. C) Expression profile of Oct4 was measured 24 h after the treatment with inhibitors in HDC. Number of Oct4-positive cells (%) from the total number of analyzed cells is indicated. Shown are data from four independent experiments treated (red) or untreated (blue) with inhibitors. Total number of cells counted per experiments 1 (exp1) and 2 (exp2) was 10,000, and in experiments 3 (exp3) and 4 (exp4) it was 100,000.

To monitor how inhibition of TGF-beta and MEK influences the expression of Oct4 and Nanog (markers of pluripotency in ESc [42]), we checked their levels in HDC before and after inhibition. Real-time PCR results showed a more than 3-fold increase of Oct4 expression 24h after the application of inhibitors (Fig 5A). In comparison, no significant increase of Oct4 expression was detected in primary skin fibroblasts or HDC of higher passage. In contrast to Oct4, we detected no significant increase of Nanog expression (when compared to its expression levels in chiPS and ESc) after treatment with inhibitors. This suggests that Nanog plays no primary role in early phases of reprogramming from HDC. Nevertheless, expression of Nanog in later phases can be clearly detected by cytoimmunofluorescence (Fig 3).

To confirm that the effect of Oct4 increase is specific to neural cells, we compared Oct4 expression with other neural specific cell lines including neurospheres and neural progenitor cell line SN4741. Similarly to HDC, 24h after addition of inhibitors, the level of Oct4 expression increased in the SN4171 line and neurospheres more than 4-fold and 2-fold, respectively (Fig 5A). Specifically, the levels of Oct4 expression in neurospheres, SN4741 and HDC reached 22%, 12% and 10.5%, respectively, of its expression in iPSc (Fig 5A). This data confirms direct effects of MEK- and TGF-inhibitors on the level of Oct4 expression in neural cells.

Finally, we wanted to determine whether only a fraction of HDC is sensitive to inhibitors. Therefore, we monitored Oct4-positive cells 24h after treatment with inhibitors. The results of the experiment indicate a statistically significant 2-fold increase of Oct4 expression in the fraction of HDC with the highest Oct4 expression compared to a nontreated control (12% versus 7%; Fig 5B). Although the significance of this increase in Oct4 expression was confirmed by four independent experiments (Fig 5C), we detected no distinct subpopulation of strongly Oct4-positive cells which would be more sensitive to inhibitor treatment but rather slight overall increase towards a higher intensity throughout the cell population. We speculate that such an elevation of gene expression could have helped only cells of specific developmental and differentiation stage to switch to embryonic program, whereas majority of other cells were unaffected regardless of Oct4 stimulation. In summary, we speculate that increase of Oct4, which controls the differentiation, can stimulate a subpopulation of developing neural cells to reprogram towards pluripotency.

### Conclusion

In this article, we demonstrate the ability to reprogram primary head-derived cell cultures into iPSc using chemical inhibition of both TGF-beta and MEK signaling pathways. Several lines of evidence point to neural cells and their progenitors as a source of cells sensitive to our reprogramming method. First, only HDC give rise to chiPS after treatment with the inhibitors. Second, we were able to detect markers for neural cells in these primary cultures (Figs B and C in S6 File). Third, expression level of Sox2 neural marker decreased during cultivation of primary cells corresponding to decreased in reprogramming efficiency (Figs A and D in S6 File). Finally, neurospheres and neural progenitor cell line SN4741 show increased Oct4 expression after inhibitor treatment similar to HDC. Accordingly, neural stem cells and derivatives of neural progenitor cells in the adult organism have been shown to be reprogrammable by exogenous overexpression of only one factor—Oct4 [43,44].

The presented results also document the existence of what might be termed “suitable” cells within a population of embryonic neural cells which can be reprogrammed towards pluripotency just by means of the chemical inhibition described. That the same reprogramming protocol did not lead to chiPSc generation from primary human fibroblasts derived from skin biopsy (data not shown) suggests that there exist different regulatory mechanisms or sample diversity. Albeit with low efficiency, the conditions described here could provide a platform upon which other genome non-integrative and safer reprogramming processes could be developed.

Research on iPSc is critical for the study of developmental biology, disease modeling, and possible applications of relatively easily accessible stem cells in research towards regenerative medicine. Therefore, understanding the mechanisms and devising improved non-integrative approaches to control cell fate and function *in vitro* and *in vivo* are crucial steps toward bringing the benefits of stem cells research into the clinic. The most recent data demonstrate that reprogramming could be achieved more easily than previously expected.

## Materials and Methods

### Ethics statement

The experiments were performed with the approval by the Ethics Committee of Faculty of Medicine, Masaryk University under valid permission from The Ministry of Agriculture of the Czech Republic, registration number CZ62760067.

### Derivation of cell lines and cultivation

Primary cell cultures were derived from the whole 7 to 15,5 DPC embryos according to the widely used protocol for feeder cells preparation [45]. Briefly, dissected cell tissues were washed with 1x PBS at 37°C and dissociated in Trypsin-EDTA for 3 min at 37°C followed by further homogenization by 1 ml pipette for 2 min. Cells were plated on 10-cm plates and cultured in MEF media consisting of knockout DMEM, 10% FBS, 1/100 (v/v) L-glutamine (200 mM), 1/100 (v/v) pen/strep, 1/100 (v/v) non-essential amino acids and 7 μl/l of β-Mercaptoethanol. After 48 h, adherent cell cultures were enzymatically passaged on PM10 plates. Primary neural cell cultures were derived using the same protocol described. Purified primary brain cells were isolated under the binocular microscope (Olympus MVX10) after surgical preparation and removal the thin layers of body tissue covering the meninges. Cells dedicated for reprogramming were seeded at concentration 25,000–33,000 cells/cm^2^. Depending on cell proliferation and density, the cells from nearly confluent plates were passed to new plates (split ratio 1:3). From day 25, MEF medium was replaced with ESM medium [36] with LIF. Replating of the cell was performed using 5 min incubation with TrypLE (Invitrogen). When required, individual forming colonies were transferred manually without enzymatic treatment.

Pluripotent epiblast stem cells (EpiSC) were isolated from dissected parts of proximal epiblasts of 7.5 DPC embryos, representing that stadium with a high number of these cells before their migration into dorsal mesentery in the region of the developing gonads [46–48], as previously described [49]. EpiSC colonies became evident over the next 4–7 days after derivation. These colonies were manually dissociated into small clusters using a glass needle and plated into 6-well plates containing feeders and medium consisting of Knockout DMEM supplemented with 20% KSR, 2 mM glutamine, 1x non-essential amino acids, 7 ml/l of ß-Mercaptoethanol, 10 ng/ml FGF2 (R&D Systems) and 10 mg/l LIF. Primordial germ cells (PGC) were derived *in vitro* from epiblast cells as described by Hayashi and coworkers [50]. For derivation of iPSc using viruses lentiviral system described previously was used [51]. Establishment of the neural progenitor cell line SN4741 was described [52]. The cell line was kindly provided by J. H. Son and grown in DMEM, 10% FCS, L-glutamine (2 mM), penicillin (50 U/ml), streptomycin (50 U/ml), glucose (0.6%) (Invitrogen). Neurosphere colonies were derived from 13.5 DPC embryonic forebrain (strain C57BL/6) and cultured in DMEM/F12 media supplemented with ITS, B27, N2 supplements, 100 i.u./ml penicillin and 0.1 mg/ml streptomycin (Invitrogene), and mouse recombinant growth factors 20 ng/ml FGF2 and 50 ng/ml EGF (Peprotech) (http://www.ncbi.nlm.nih.gov/pubmed/15289455). Two- to four-day primary neurospheres were used in experiments.

### Inhibitors

Inhibitor of ALK5 (SB431412), MEK (PD0325901) and p53 (Cyclic Pifithrin-alpha), were purchased from Stemgent. Working concentration was 0.5 μM for PD0325901, 2 μM for SB431542 and 20 μM for Cyclic Pifithrin-alpha. MicroRNA mimics were purchased from Sigma-Aldrich and transfected according to manufacturer’s protocol using FuGene HD transfection reagent (Roche) at days 1, 3, and 7 in 120 nM concentration, based on previous publication [29].

### Immunostaining

For immunofluorescence analysis the chiPSc (chemically induced pluripotent stem cells) and PGCs were fixed with 4% paraformaldehyde, washed three times with phosphate-buffered saline (PBS) and permeabilized by 1xPBS with 0.1% Triton X-100 and 1 mg/ml bovine serum albumin. Primary antibody incubation was carried out overnight at 4°C, followed by three washes in PBS and incubation with individual secondary antibodies (Alexa 564, Alexa 488; Molecular Probes, Eugene) for 1 h at room temperature, and washed in PBS. The following antibodies and corresponding dilutions were used: Oct4 (ab19857) 1:100, Nanog (ab80892) 1:400, Sox2 (ab59776), Ssea1 (ab16285) 1:200, Ddx4 (ab13840) 1:100, Fragilis (ab15592) 1:100, Stella (ab19878) 1:125. Nuclei were counterstained with DAPI and then mounted in Mowiol (Polysciences, Warrington, PA) containing 1,4-diazobicyclo-[2.2.2.]-octane to prevent fading. For staining of neural cells primary antibodies against Tuj1 (ab14545; 1:1000), Nestin (Ab27952; 1:300), and Gata4 (ab84593; 1:75) were used. Microscopic analysis was performed using an Olympus FluoView 500 laser-scanning microscope. Alkaline phosphatase (VectorRed alkaline Cat. No. SK-5100) staining was performed according to the manufacturer’s protocol.

### Teratoma formation assay and neural cell differentiation

The cells from two chiPSc lines were harvested by collagenase IV treatment, collected into tubes, centrifuged, and the pellets suspended in DMEM/F12. One-third of the cells (2 million) from a confluent 10-cm plate was injected subcutaneously into the muscle of the hind leg of CF-1 mouse strain. Four weeks after injection, tumors were dissected, weighted, and fixed with PBS containing 10% PAF solution at room temperature for 12 h. Paraffin-embedded tissue was sliced, stained with hematoxylin and eosin and analyzed. Neural differentiation was induced as described previously [31, 17]. Briefly, chiPS cells were plated onto 0.1% (v/v) gelatin-coated tissue culture plastic at 1×10^4^ cells/cm^2^ in N2B27 neurobasal media (with 50% DMEM/F12 (Invitrogen)) in serum-free conditions supplemented with and 100U/ml LIF (Millipore). From day 2, medium was supplemented with 5 ng/ml bFGF (Peprotech).

### Fluorescence Activated Cell Sorting (FACS)

Cell cultures reaching ~80% confluence were washed and dissociated to obtain single cell suspensions using Trypsin/EDTA (Sigma T3924) + 2% chick serum. Cells were washed again with 1x PBS (w/o Ca/Mg++) + 2% FBS + 0.1% NaN3 and dissociated by gentle pipetting. Wash steps were followed by centrifugation for 5 minutes at 200g and cell pellet was resuspended in 1ml of 1x PBS with paraformaldehyde in final concentration 0.5% and fixed for 10 min in 37°C water bath. After centrifugation, cells were washed with 1x PBS (w/o Ca/Mg++) + 2% FBS + 0.1% NaN3 and resuspended in ice-cold methanol (90%) for 30 minutes. After washing, the supernatant was purred off and add 100μl of pre-diluted primary mouse monoclonal antibodies against Oct3/4 (sc-5279; Santa Cruz Biotechnology) were added at 1:50 (v/v). After overnight incubation at 4°C cells were washed 1x PBS (w/o Ca/Mg++) + 2% FBS + 0.1% Triton and incubated with Alexa Fluor 488 goat anti-mouse IgG2a (1:500 dilution, Invitrogen A21131). A Becton Dickinson FACSAria instrument was calibrated before each experiment with the negative gate set using isotype controls. Non-viable cells were excluded using 0.1% v/v propidium iodide (SIGMA P4864).

### RNA extraction and Real-time PCR analysis

Total RNA from analyzed cells was extracted with a High pure RNA isolation kit (Roche 11828665001) according to the manufacturer’s instructions. RNA concentration and purity were determined using a NanoDrop-1000 spectrophotometer (Thermo Scientific). Total RNA (200–500 ng) was reverse transcribed using the Transcriptor first strand cDNA synthesis kit (Roche; 04897030001) according to the manufacturer’s instructions. Equal volumes of cDNA reaction were added to the Real-time PCR reactions. Target cDNA levels were analyzed by the comparative cycle time (Ct) method of Real-time quantitative RT–PCR with 20 μl reactions performed with LightCycler 480 SYBR Green I Master Mix (Roche; 04707516 001) and gene specific primers (Oct4 F:AAGCGATCAAGCAGCGACTAT, R:GGAAAGGGACCGAGGAGTACA, Nanog F: CAAAGGCAAACAACCCACTT, R: TCTGCTGGAGGCTGAGGTAT and GAPDH F:TCATTTCCTGGTATGACAACG, R:ATGTGGGCCATGAGGT). Triplicate assays were carried out and the mean relative level of expression and associated standard deviations were calculated using advanced relative quantification method in Lightcycler 480 II software (Roche).

### Karyotyping

Actively growing ES cells were treated with Demecolcin (20 μl of 10 μg/ml in 10 ml of media) for 1.5 hour. After detaching of cells with 1xPBS with Accumax (Millipore), cell suspension was incubated at 37°C in 0.56% KCl for 20 min. Cells were fixed with methanol: acetic acid 3:1 (Sigma), dropped onto glass slide and air dried. After the staining with Orcein (Sigma; one drop per slide), chromosomes were counted using microscope with phase contrast and 40x magnification. A total number of 20–40 good spreads were analyzed in four chiPS lines. For G-banding, slides with chromosomal spreads were incubated for 30 min at 80°C. After 5–7 sec digestion in 2% Trypsine (BD Difco) in Sorensen’s phosphate buffer (0.133 M Na2HPO4, 0.133 M KH2PO4, pH 6,8) chromosomes were stained for 2 min in Giemsa-Romanovski staining solution (5% Giemsa-Romanovski in Sorensen’s phosphate buffer). After the washing with distilled water and air-drying, 20–30 good mitotic spreads were captured using microscope under 100x magnification and karyotype was analysed using Lucia Karyo software (Lucia cytogenetics; http://www.lucia.cz).

### Statistical analysis

In pertinent experiments, differences in the levels of expression were evaluated for statistical significance according to P values gained from a two-tailed Student's t-test with a 95% confidence interval using Microsoft Excel 2010.

## Supporting Information

S1 File Karyotypic analysis of chiPS. G-banded karyotype from chiPS cell at passage 5 retaining normal number of chromosomes 2x = 40. Total number of 20–30 good mitotic spreads and karyotypes were analyzed in 4 different chiPS lines. Magnification 100x.
(TIF)

S2 File Tuj1 and Nestin Expression during Differentiation. Tuj1-positive (A; green) and Nestin-positive cells (B; red) detected eight days after the induction of neural differentiation of chiPS. Overlay with DAPI (blue). Elongated structures of neuronal dendrites and cell heterogeneity is visible (C; phase contrast image). Magnified 20x.
(TIF)

S3 File Fibroblast cell cultures during first 10 days of reprogramming. Primary cell cultures derived from neuron-rich head samples from 12,5 DPC lines. Significant “homogenization” of cells culture is visible during first period of reprogramming. Neural cells which are abundant in picture from day 1 and day 3 significantly disappear during the cell culture growth and passaging. Light microscopy photo; size bars in [μm] are indicated.
(TIF)

S4 File Efficiency of primary cell cultures to reprogramming. Primary cell cultures derived from different parts of the body were examined for reprogramming efficiency. Number of alkaline-phosphatase-positive (AP) colonies was counted per 1.7 x 10^6^ seeded cells from four independent experiments (Fig A). The reprogramming efficiency for HDC cultures derived from 8 to 15.5 DPC embryos and adult mouse per 1.7 x 10^6^ seeded cells from three independent experiments (Fig B).
(DOCX)

S5 File Immunostaining of mouse embryonic stem cells (mES), primordial germ cells (PGC), and chemically- (chiPS) or virally- (viPS) derived mouse iPSc. The expression of specific pluripotent stem cells and germ cells markers including Nanog, Stella (Dppa3) and Fragilis (Iftm3) together with specific germ cells marker Ddx4 (Mvh; mouse vasa homolog) in chiPS, iPS, ESc and PGC cells. Nuclei were stained using DAPI (in blue).
(TIF)

S6 File Characterization of primary cell culture. Efficiency of reprograming decreases with time in cultivation. Application of the same reprogramming protocol on primary cells which were cultivated three weeks in-vitro, resulted in complete loss of reprogramming capacity of these cells (Fig A). Immunostaining for Gata4, Nestin, and Tuj1 in the samples of HDC. No positive signal was detected in samples of primary skin fibroblasts (Fig B). Counterstaining with DAPI (blue) is also shown. Number of cells positive for neural markers were counted using image analysis from three independent experiments (Fig C). Real-time PCR analysis of Sox2 expression level during cultivation (Fig D). As a reference GAPDH was used. Two weeks of cultivation of HDC the level of Sox2 is significantly reduced. Data normalized to the level of Sox2 expression at day 0; 100% (after derivation of in-vitro cell culture) and represent average ± s.d. from three independent experiments. *P<0.05. NS, non-significant P>0,05, statistical analysis performed by one-tail paired t-test.
(TIF)
