# Supplementary figures and images for: MEK and TGF-beta Inhibition Promotes Reprogramming without the Use of Transcription Factor
Source: PLoS One. 2015 Jun 3;10(6):e0127739. doi: 10.1371/journal.pone.0127739 (PMC4454598; doi:10.1371/journal.pone.0127739)

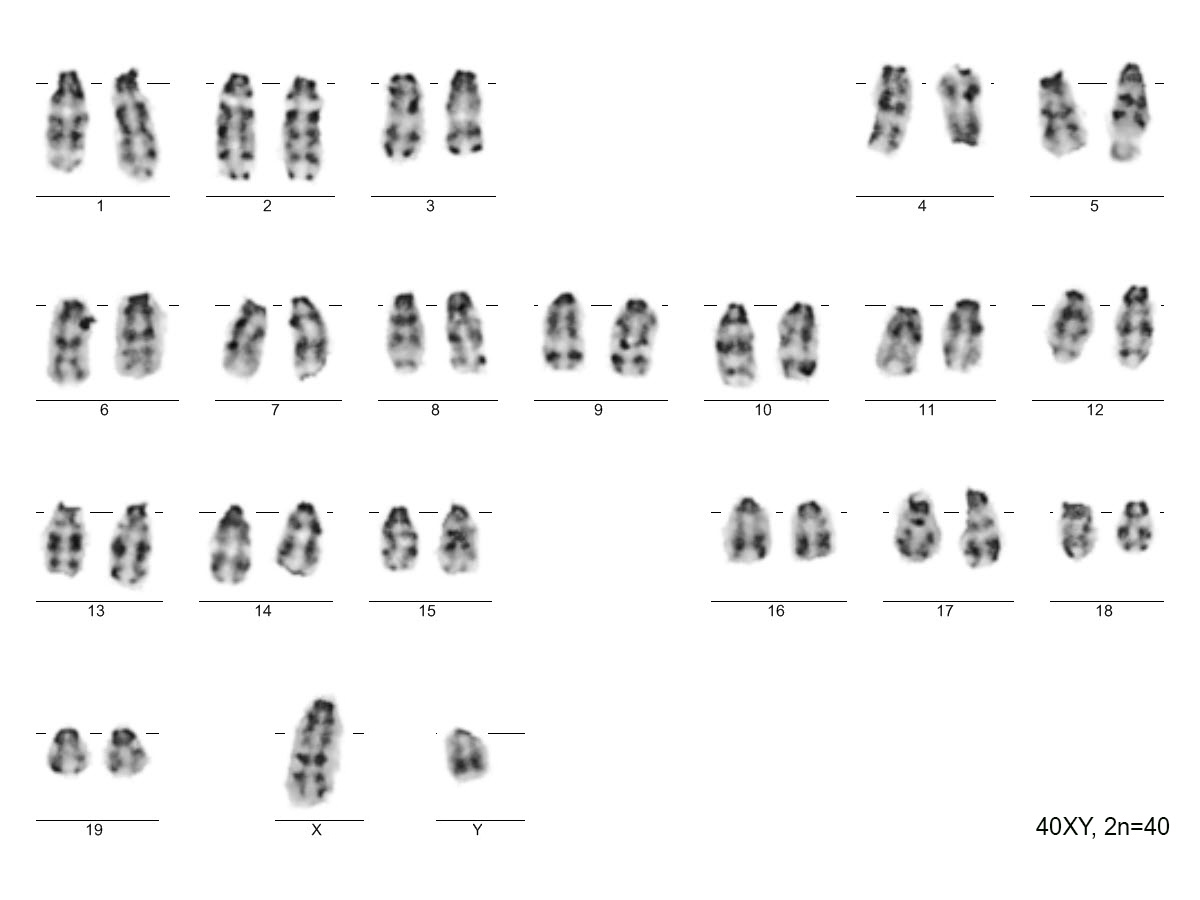

Supplement: S1 File — G-banded karyotype from chiPS cell at passage 5 retaining normal number of chromosomes 2x = 40. Total number of 20–30 good mitotic spreads and karyotypes were analyzed in 4 different chiPS lines. Magnification 100x. (TIF) [file pone.0127739.s001.tif]

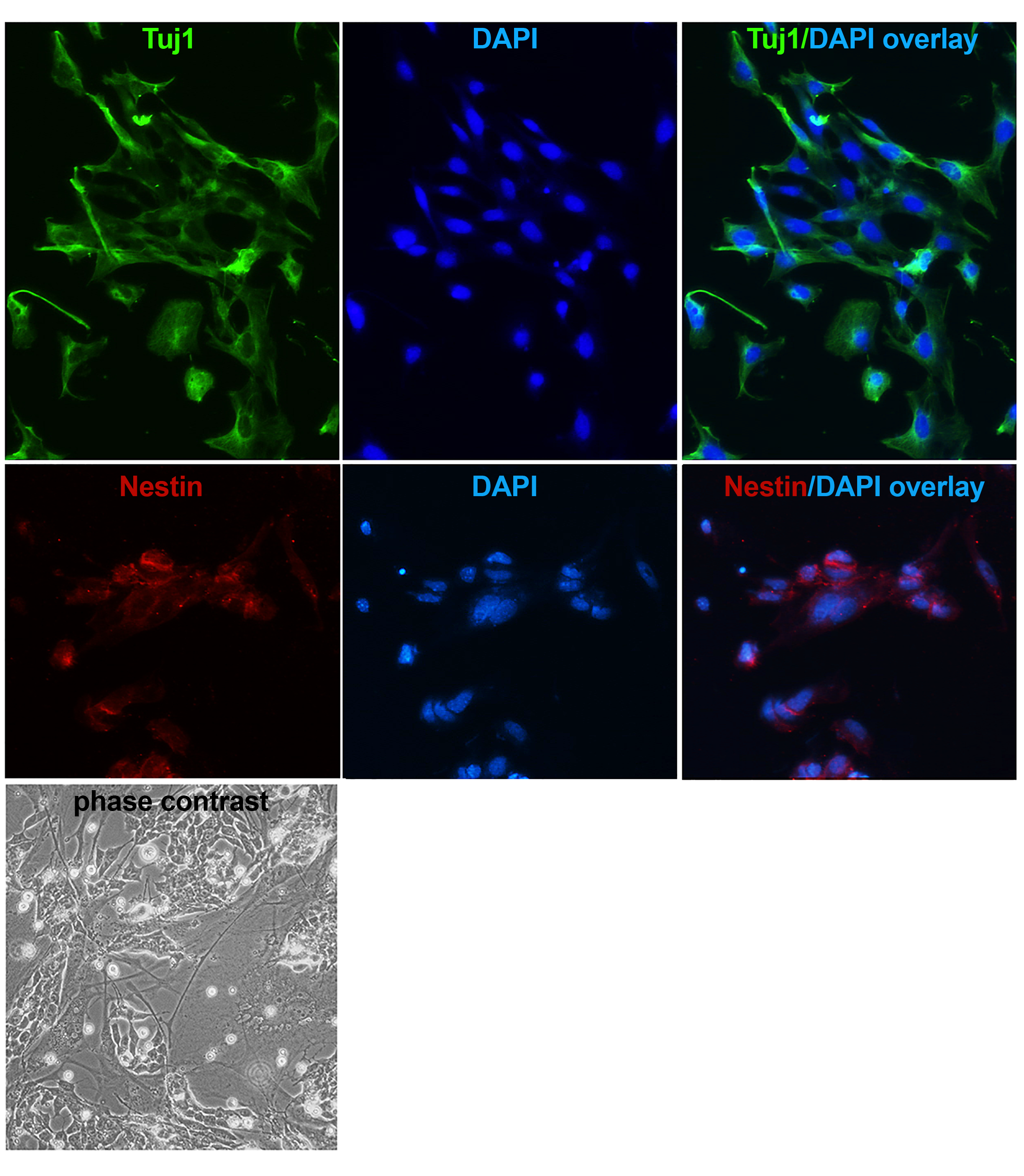

Supplement: S2 File — Tuj1-positive (A; green) and Nestin-positive cells (B; red) detected eight days after the induction of neural differentiation of chiPS. Overlay with DAPI (blue). Elongated structures of neuronal dendrites and cell heterogeneity is visible (C; phase contrast image). Magnified 20x. (TIF) [file pone.0127739.s002.tif]

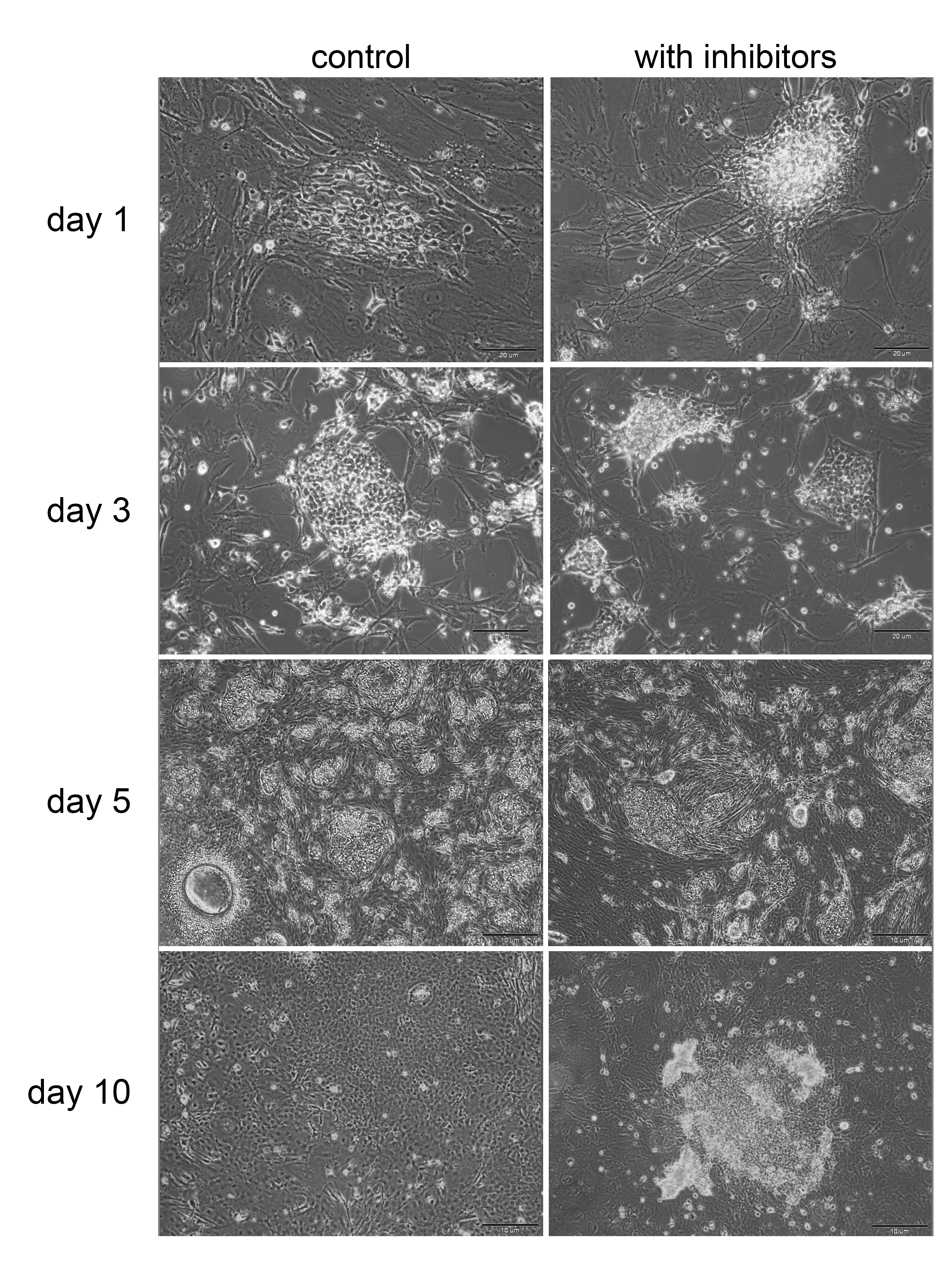

Supplement: S3 File — Primary cell cultures derived from neuron-rich head samples from 12,5 DPC lines. Significant “homogenization” of cells culture is visible during first period of reprogramming. Neural cells which are abundant in picture from day 1 and day 3 significantly disappear during the cell culture growth and passaging. Light microscopy photo; size bars in [μm] are indicated. (TIF) [file pone.0127739.s003.tif]

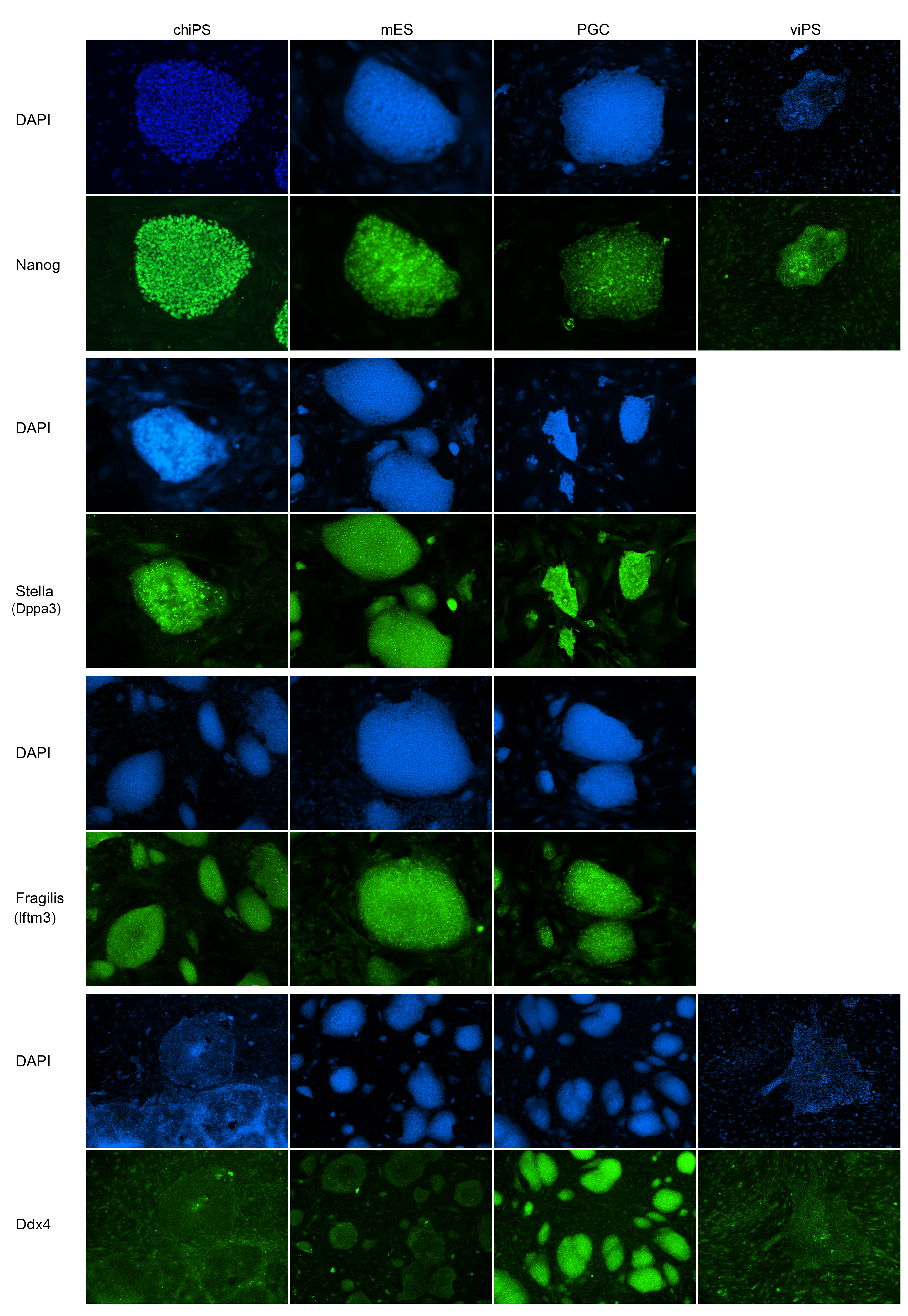

Supplement: S5 File — The expression of specific pluripotent stem cells and germ cells markers including Nanog, Stella (Dppa3) and Fragilis (Iftm3) together with specific germ cells marker Ddx4 (Mvh; mouse vasa homolog) in chiPS, iPS, ESc and PGC cells. Nuclei were stained using DAPI (in blue). (TIF) [file pone.0127739.s005.tif]

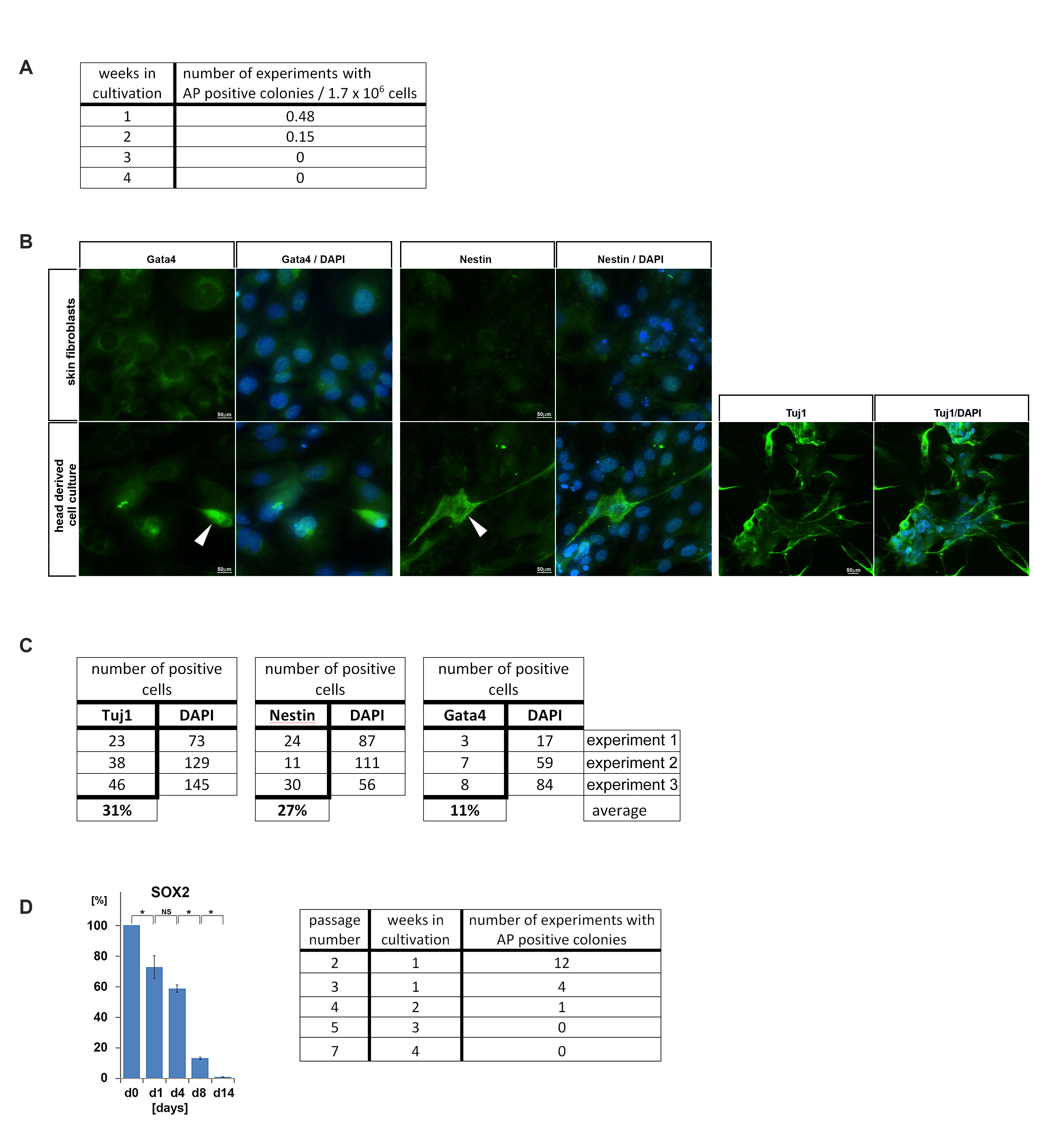

Supplement: S6 File — Efficiency of reprograming decreases with time in cultivation. Application of the same reprogramming protocol on primary cells which were cultivated three weeks in-vitro, resulted in complete loss of reprogramming capacity of these cells (Fig A). Immunostaining for Gata4, Nestin, and Tuj1 in the samples of HDC. No positive signal was detected in samples of primary skin fibroblasts (Fig B). Counterstaining with DAPI (blue) is also shown. Number of cells positive for neural markers were counted using image analysis from three independent experiments (Fig C). Real-time PCR analysis of Sox2 expression level during cultivation (Fig D). As a reference GAPDH was used. Two weeks of cultivation of HDC the level of Sox2 is significantly reduced. Data normalized to the level of Sox2 expression at day 0; 100% (after derivation of in-vitro cell culture) and represent average ± s.d. from three independent experiments. *P<0.05. NS, non-significant P>0,05, statistical analysis performed by one-tail paired t-test. (TIF) [file pone.0127739.s006.tif]
