# Supplementary material for: MEK and TGF-beta Inhibition Promotes Reprogramming without the Use of Transcription Factor
Source: PLoS One. 2015 Jun 3;10(6):e0127739. doi: 10.1371/journal.pone.0127739 (PMC4454598; doi:10.1371/journal.pone.0127739)
Supplement: S4 File — Primary cell cultures derived from different parts of the body were examined for reprogramming efficiency. Number of alkaline-phosphatase-positive (AP) colonies was counted per 1.7 x 10^6^ seeded cells from four independent experiments (Fig A). The reprogramming efficiency for HDC cultures derived from 8 to 15.5 DPC embryos and adult mouse per 1.7 x 10^6^ seeded cells from three independent experiments (Fig B). (DOCX) [file pone.0127739.s004.docx]

**S4 File**

**A B**

| Cell sample | number of AP positive colonies  in subsequent experiments |  | Age of the cells [DPC] | number of AP positive colonies  in subsequent experiments |
| --- | --- | --- | --- | --- |
| whole embryo | 3 / 1 / 7 / 0 |  | 8 | 2 / 4 / 0 |
| liver | 0 / 0 / 0 / 0 |  | 10 | 3 / 1 / 6 |
| side-body skin | 0 / 0 / 0 / 0 |  | 11,5 | 0 / 9 / 5 |
| head | 2 / 4 / 0 / 10 |  | 12,5 | 2 / 5 / 8 / 20 |
| brain | - |  | 13,5 | 6 / 2 / 1 |
| tail-tip | 0 / 0 / 0 / 0 |  | 14,5 | 0 / 0 / 0 |
|  |  |  | 15,5 | 0 / 0 / 0 |
|  |  |  | Adult mouse | 0 / 0 / 0 |
